# Cannabinoids: Therapeutic Perspectives for Management of Orofacial Pain, Oral Inflammation and Bone Healing—A Systematic Review

**DOI:** 10.3390/ijms26083766

**Published:** 2025-04-16

**Authors:** Maria Domenica Campana, Giulio de Paolis, Gilberto Sammartino, Paolo Bucci, Angelo Aliberti, Roberta Gasparro

**Affiliations:** Department of Neuroscience, Reproductive Science and Dentistry, University of Naples Federico II, 80138 Naples, Italy; mariadomenica.campana@unina.it (M.D.C.); g.depaolis@studenti.unina.it (G.d.P.); angelo.aliberti@studenti.unina.it (A.A.); roberta.gasparro@unina.it (R.G.)

**Keywords:** cannabinoids, THC, CBD, terpenes, pain management, bone regeneration, oral surgery, dentistry

## Abstract

Cannabinoids, particularly cannabidiol (CBD) and tetrahydrocannabinol (THC), have been increasingly studied for their therapeutic applications in various medical fields. This systematic review aims to explore their role in oral surgery, focusing on pain management, inflammation control, and bone regeneration. A systematic review was conducted using the PRISMA framework to identify relevant studies from the PubMed, Scopus, and Web of Science databases published up to November 2024. The review included clinical and preclinical studies investigating the effects of cannabinoids on orofacial pain, oral inflammation, and bone healing. Data on study design, cannabinoid types, and relevant outcomes were extracted and analyzed. CBD was the most commonly studied compound, with other studies evaluating CB1/CB2 receptor agonists, THC, and cannabis smoke. Clinical trials showed mixed results: some studies found CBD effective in reducing dental or myofascial pain, while others found limited or non-superior outcomes compared to standard treatments (e.g., NSAIDs, corticosteroids). Among the four RCTs, three had a low risk of bias, and one moderate; all nine animal studies had a high risk of bias. in conclusion, preclinical and clinical studies suggest that cannabinoids represent a promising non-opioid alternative for pain management and for oral inflammation. Although some evidence suggests potential benefits of cannabinoids, particularly CBD, in oral health contexts, findings are derived from heterogeneous studies—many with high risk of bias. More high-quality, standardized clinical trials are necessary before recommending cannabinoids for routine dental practice.

## 1. Introduction

**Figure 1:**
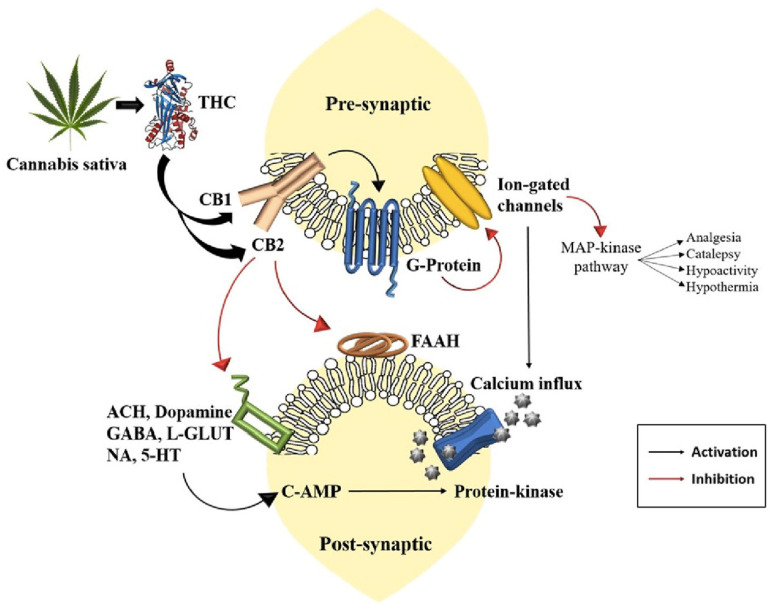
Mechanism of action of THC [6].

Oral and implant surgeries are commonly associated with significant postoperative sequelae, including pain, inflammation, and delayed bone healing. These complications can negatively affect patient recovery and the overall success of the surgical intervention. Traditional approaches to pain management, such as opioids and nonsteroidal anti-inflammatory drugs (NSAIDs), are effective but have notable limitations, including the risk of dependency, gastrointestinal side effects, and inadequate long-term efficacy [1]. Consequently, there is an increasing need for safer and more effective alternatives. Cannabinoids, particularly cannabidiol (CBD) and tetrahydrocannabinol (THC), have shown considerable potential in addressing these challenges [2]. Their therapeutic effects are mediated through the endocannabinoid system (ECS), which plays a key role in regulating pain, inflammation, and immune responses. Specifically, CB1 and CB2 receptors are involved in modulating neuronal transmission and immune activity, respectively (Figure 1) [3,4,5].

Cannabis oil, recognized for its antioxidant properties, may help mitigate oxidative damage by scavenging ROS and upregulating antioxidative mechanisms, potentially enhancing wound healing. Additionally, CBD has been shown to influence non-cannabinoid pathways, such as the TRPV1 and serotonin receptors, further contributing to its analgesic and anti-inflammatory properties [7,8]. Beyond these effects, the “entourage effect”, which describes the synergistic interaction between cannabinoids, terpenes, and flavonoids, enhances the therapeutic potential of these compounds. For instance, flavonoids like quercetin and cannflavin amplify the anti-inflammatory and analgesic properties of cannabinoids [2]. Terpenes such as beta-caryophyllene also play a crucial role, acting as agonists of CB2 receptors to reduce inflammation and pain [2,8]. The selective activation of the CB2 receptor could represent a new therapeutic strategy to reduce osteoclastic activity and bone resorption in patients with Paget bone disease. This approach could also help manage the pain associated with the disease. Treatment with JWH-133, a selective CB2 agonist, resulted in a significant reduction in the number and activation of osteoclasts. On the other hand, the TRPV1 receptor agonist increased osteoclast activity, while its antagonist, I-RTX, led to a decrease in osteoclast activation. In terms of bone resorption activity, CB2 activation with JWH-133 effectively reduced the area of bone resorption, highlighting its potential to control excessive bone degradation. Conversely, the use of the CB2 inhibitor (AM630) resulted in increased bone resorption, further illustrating the regulatory role of CB2 in bone remodeling. However, further studies are needed to validate these results in larger samples [9,10]. CBD acts on bone fracture healing through various mechanisms. First, it stimulates mesenchymal stem cells, which can differentiate into osteoblasts, thereby improving the formation of bone tissue during the fracture healing process. This accelerates bone regeneration, increasing the mechanical strength of the newly formed bone. Additionally, CBD appears to enhance the biomechanical quality of the newly formed bone in association with the increased expression of procollagen-lysine 2-oxoglutarate 5-dioxygenase (PLOD1), making the healed fracture more resistant. However, no significant impact on bone health has been observed in the absence of fractures or damage, suggesting that CBD has a targeted effect mainly when bone repair is needed [11,12].

This encourages the idea that after an implant procedure, a protocolled therapeutic assumption of cannabinoids, terpenes, and flavonoids, might enhance the healing time and increase the osteointegration. While preclinical studies have provided robust evidence for the efficacy of cannabinoids in managing pain and inflammation, as well as promoting bone regeneration, clinical results have been more variable. Furthermore, several literature reviews have explored the use of cannabidiol for oral health, but most have focused primarily on the number of cannabinoid receptors in different dental conditions, without demonstrating the specific effects of these compounds on such conditions [13,14,15]. Thus, this systematic review aims to synthesize the current literature on the effect of cannabinoids, focusing on their analgesic and anti-inflammatory properties in bone healing. By examining their mechanisms of action and therapeutic synergies, this study seeks to evaluate their potential for improving outcomes in dentistry.

## 2. Materials and Methods

A systematic review was conducted using the PRISMA framework [16]. According to the PICO statement, this systematic review aimed to answer to the question: is the use of cannabinoid drugs (intervention) effective for the management of orofacial pain (primary outcome) in dental patients (population)? Oral lesions and bone healing have been considered as secondary outcomes. Other drugs instead of cannabinoids were used as comparisons. This systematic review was registered on the PROSPERO database (number CRD420250652264). No deviations from the initial protocol were encountered.

### 2.1. Literature Search

Articles were searched in PubMed, Scopus, and Web of Science up to November 2024 using the MeSH Terms and keywords listed in Table 1. The software Rayyan (online free program, Cambridge, MA, USA) was used to select articles in duplicate by two blinded authors (MDC, GDP).

A manual search was performed directly from the websites of the following scientific journals: *Pharmacology*, *Pharmacological Research*, *Clinical Pharmacology and Therapeutics*, *Dentistry Journal*, *Biodrugs*, and *Trends in Pharmacological Sciences*. Two authors (MDC, GDP) separately carried out the electronic literature search. After title and abstract screening, the articles were selected for full-text reading. Whenever differences in the judgment of the eligibility of title and abstract occurred, full texts were included for final assessment. All RCT studies, CCTs, cross-sectional studies, case reports, case series, animal studies published until November 2024 about the effects of CBDs on orofacial pain, bone healing after oral surgery and pain linked to soft tissue oral lesions compared to other drugs, have been included. Questionnaires, literature reviews, systematic reviews, and all studies on patients suffering from oncological or psychiatric pathologies were excluded. Articles written in any language other than English were excluded. To identify unpublished or discontinued studies, all authors of the selected studies were contacted and the bibliographies of all selected studies and relevant reviews were checked. Disagreements between the two investigators were solved through discussion; if needed, a third operator (RG) was contacted for final decision.

### 2.2. Data Extraction

Data were independently extracted by two authors (MDC, GDP) using a pre-determined extraction form. The following data were extracted: publication year, country of origin, type of study design and sample size, type of cannabinoid used (CBD, THC, or others), outcome measures related to pain, inflammation and bone healing, details about the results obtained (including methods of measurement and time intervals), complication rates.

### 2.3. Risk of Bias Assessment

Two authors (AA and GS) independently assessed the studies in terms of inclusion criteria, relevance, eligibility, and risk of bias following the recommendations of the Joanna Briggs Institute Critical Appraisal tool (JBI) [17] for randomized-controlled trials, SYRCLE’s risk of bias [18] for animal studies and QUIN (Quality Assessment Tool for In Vitro Studies) [19] for in vitro studies in dentistry. Any disagreement was solved by consensus between reviewers and statisticians (PD). The JBI does not provide a range of scores that indicate the overall quality, but considering the relative importance of each domain and its potential impact on the study results and interpreting the domain scores in the context of the study, the studies were classified as having a low risk of bias if most domains score “Yes”, a moderate risk if some domains are rated “No/Unclear”, and a high risk if multiple domains have significant bias or are rated “No”. The SYRCLE’s tool addresses the following types of biases: selection bias, performance bias, attrition bias, detection bias, and reporting bias. A “yes” judgment indicates a low risk of bias; a “no” judgment indicates a high risk of bias; the judgment will be “unclear” if insufficient details have been reported to assess the risk of bias properly. Finally, the QUIN tool consisted of 12 criteria (clearly stated aims/objectives, detailed explanation of sample size calculation, detailed explanation of sampling technique, details of comparison group, detailed explanation of methodology, operator details, randomization, method of measurement of outcome, outcome assessor details, blinding, statistical analysis, and presentation of results) that can be evaluated as adequately specified = 2 points, inadequately specified = 1 point, not specified = 0 point, and not applicable = exclude criteria from calculation. The scores were then added to obtain a total score for a particular in vitro study. The scores thus obtained were used to grade the in vitro study as high-, medium-, or low-risk (>70% = low risk of bias, 50% to 70% = medium risk of bias, and <50% = high risk of bias) by using the following formula: final score = (total score × 100)/(2 × number of criteria applicable).

## 3. Results

### 3.1. Selection of Studies

**Figure 2:**
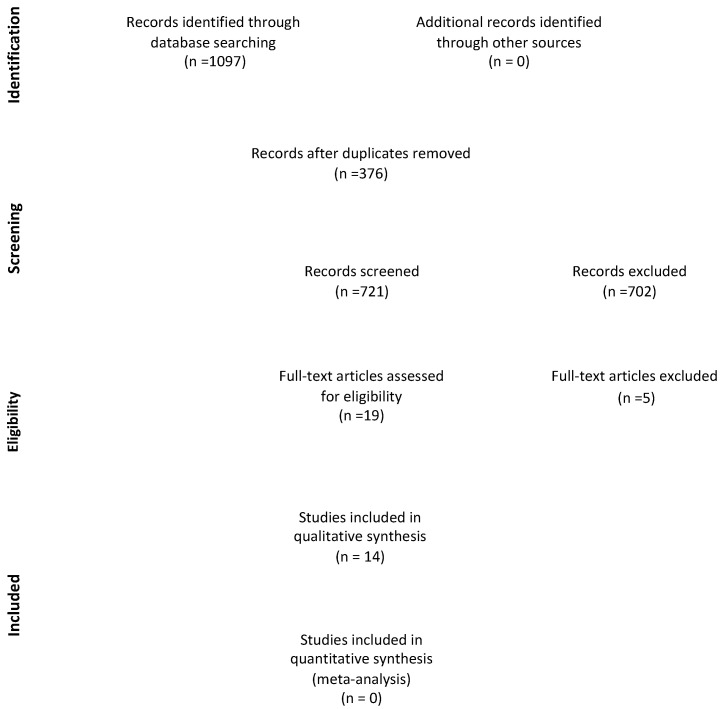
PRISMA flow diagram.

In total, the initial search strategies generated 1097 articles. After duplicate removal, 721 articles remained for title and abstract evaluation. A total of 702 papers were excluded due to mismatch with our search criteria, different evaluated outcomes, and topic. Then, 19 articles were retained for final full text review. Finally, 14 articles [20,21,22,23,24,25,26,27,28,29,30,31,32,33] which have so far evaluated the potentially of cannabinoid on orofacial pain, oral inflammation, and bone healing. Reports were excluded if they were narrative reviews, systematic reviews, correspondence, and letters to the editor, as shown in Table 2 [34,35,36,37,38,39]. The results of the selected studies were classified under the following subheadings: effect of cannabinoid on orofacial pain, oral inflammation, and bone healing (in vitro or in vivo). Some studies touched upon different characteristics; therefore, they were included in two/three categories called “synergistic effects of cannabinoids”. The Preferred Reporting Items for Systematic Reviews and Meta-Analyses (PRISMA) Flow Diagram 2020 in Figure 2 depicts the flow of included studies through each phase of the review process.

### 3.2. Subgroup Analysis

To explore potential variability in outcomes and strengthen the validity of the findings, a subgroup analysis was performed based on the type of condition treated (orofacial pain, oral inflammation, and bone healing), [Table 3] type of cannabinoid used (CBD, THC, and CB2 receptor agonists), [Table 4] study model (preclinical vs. clinical), [Table 5] and mode of administration (topical, oral, or injectable formulations) [Table 6].

### 3.3. Clinical Results

The main results of the study are reported in Table 7.

#### 3.3.1. Analgesic Effect/Oral Ulcers Therapy/Oral Gingivostomatitis Therapy

Chrepa, V. and collaborators [21] investigated the effectiveness and safety of cannabidiol (CBD) as a non-opioid analgesic for emergency dental pain. They showed that CBD significantly reduced pain scores compared to the placebo. CBD at 10 mg/kg (CBD10) and 20 mg/kg (CBD20) doses provided maximum pain relief within 180 min of administration. CBD20 achieved faster onset of significant pain relief (15 min) than CBD10 (30 min). Moreover, both CBD doses significantly increased bite force, indicating improved masticatory function. However, CBD caused minimal adverse effects including sedation (calmness or relaxation), diarrhea, and abdominal pain, which resolved quickly or with minor interventions. In the study of Umpreecha, C. [33], the authors investigated the safety and efficacy of topical 0.1% cannabidiol (CBD) for managing recurrent aphthous ulcers. No allergic reactions, side effects, or changes in vital signs or blood parameters occurred after CBD use. CBD reduced ulcer size more significantly than placebo at all time points. CBD reduced erythematous size significantly by day 2 and reduced pain compared to placebo by day 5, concluding that topical 0.1% CBD is safe and effective, demonstrating anti-inflammatory effects in early RAU stages and analgesic effects in later stages. Thus, CBD could be an alternative for RAU patients who prefer not to use steroids, except in cases where CBD is contraindicated. As the same way, Qi, X. et al. [31] showed that CBD oral spray on induced oral ulcers on mice tongue inhibits inflammation, relieves pain, and accelerates lesion closure. Coelho [22] assessed the clinical efficacy and safety of a commercially available cannabidiol (CBD) oral formulation as an adjunctive pain management treatment for feline chronic gingivostomatitis (FCGS). CBD significantly improved disease severity in cats with FCGS, and the treatment was safe, with no major adverse effects. Ostenfeld [31] evaluated the postoperative pain-relief efficacy of GW842166, a noncannabinoid CB2 agonist, in patients undergoing a third molar extraction. Patients received either GW842166 (100 mg or 800 mg), ibuprofen (800 mg initially, followed by 400 mg after 4 h), or placebo preoperatively. GW842166 (at both 100 mg and 800 mg doses) did not provide meaningful postoperative pain relief compared to ibuprofen or placebo in acute dental pain scenarios. Ibuprofen remained superior across all evaluated endpoints. Nitecka-Buchta, A. and collaborators [27] showed that the application of CBD formulation over masseter muscle reduced the activity of masseter muscles and improved the condition of masticatory muscles in patients with myofascial pain.

#### 3.3.2. Anti-Inflammatory Effect

Five studies [20,23,25,28,29] evaluated the anti-inflammatory effect of CBD, all in induced periodontitis in rats.

Ossola, C. and collaborators [28] evaluated the effects of the CB2 receptor agonist HU-308 on oral health in rats with induced periodontitis, applied topically to the gingiva daily. The findings showed that HU-308 exhibited anti-inflammatory, bone-protective, and pro-homeostatic effects in rats with induced periodontitis. These findings suggest potential therapeutic benefits of CB2 receptor agonists for managing periodontal disease. As before, the same authors [29] assessed the effect of long-term treatment with the synthetic cannabinoid methanandamide (Meth-AEA) on the progression of periodontitis in rats. They showed that cannabinoid significantly diminished the alveolar bone loss, compared to rats without treatment. The treatment also reduced the production of some biological mediators of periodontal disease such as tumor necrosis factor alpha and nitric oxide. Also, Se Woong Kim [23] examined the combined effects of CBD and taurine on inflammatory markers and periodontitis in rats. In rats with periodontitis, treatment with CBD and taurine significantly reduced alveolar bone resorption, periodontal pocket depth, and the distance between the cementoenamel junction (CEJ) and the alveolar bone crest (ABC), suggesting that their combination could be a novel treatment for periodontal disease. According to previous results, Napimoga, M. [25] tested the effects of CBD in a periodontitis experimental model in rats. Morphometrical analysis of alveolar bone loss demonstrated that CBD-treated animals presented a decreased alveolar bone loss and a lower expression of the activator of nuclear factor-κB ligand RANKL/RANK, along with lower interleukin (IL)-1β and tumor necrosis factor (TNF)-α production. These results indicate that CBD may be useful to control bone resorption during the progression of experimental periodontitis in rats. In the end, Chen, H. [20], investigated CBD’s anti-inflammatory effects and its potential to protect bone in periodontitis. CBD significantly reduced bone loss in the experimental periodontitis model, downregulated TNF-α, and reduced TLR4 expression in gingival tissues, suggesting that CBD, particularly in topical form, could be an effective therapeutic agent for treating periodontitis.

#### 3.3.3. Bone Healing/Regeneration Effect

Qi, X. and collaborators [32] investigated the potential of cannabidiol (CBD) to promote odonto/osteogenesis in human dental pulp cells (HDPCs) for applications in vital pulp therapy. CBD demonstrated bi-phasic effects on HDPC viability, enhancing proliferation and cell migration. Moreover, CBD enhanced collagen synthesis (types I and III) and mineral deposition and increased expression of odonto/osteogenic markers, suggesting that CBD has therapeutic potential as a biocompatible agent for enhancing pulp repair and regeneration in dentistry. Nogueira-Filho [27] investigated the effects of Cannabis sativa (marijuana) smoke on bone healing around titanium implants in rats, exposed to marijuana smoke for 8 min daily. The authors showed deleterious effect on bone healing in the cancellous bone surrounding titanium implants, potentially raising concerns for implant success in marijuana users. According to the previous study, Klein, K. and collaborators [24] also showed that marijuana, through its active component tetrahydrocannabinol (THC), affects bone remodeling in rats undergoing orthodontic tooth movement.

### 3.4. Risk of Bias Results

The results of the bias risk assessment for randomized controlled trials (RCTs) included in the review are shown in Table 8. Only four RCTs were included in the article. Most domains were satisfied; instead, only one domain (“Were outcomes assessors blind to treatment assignment?”) scored “unclear” for one RCT. Despite that, all RCTs resulted as low risk of bias. Nine animal studies were included in the article. The most satisfied domains were selection, performance, and detection bias. Overall, according to SYRCLE’S risk of bias, animal studies included were assessed as having a high risk of bias (Table 9). Finally, only one in vitro study was evaluated as having a low risk of bias according to the nine criteria recognized as applicable (Table 10).

## 4. Discussion

This systematic review aims to synthesize the current literature on the effect of cannabinoids, focusing on their analgesic, anti-inflammatory, and properties for bone healing. Cannabinoids, the active compounds found in the cannabis plant, have gained increasing attention in medical science due to their potential therapeutic benefits. The clinical importance of cannabinoids lies in their ability to modulate a range of medical conditions, particularly those for which conventional treatments may be limited or ineffective. Cannabinoids interact with the endocannabinoid system (ECS) to modulate pain and inflammation, with CBD showing unique advantages through its interaction with TRPV1 and serotonin receptors [40]. These mechanisms align with existing evidence and support CBD as a safer alternative to opioids and NSAIDs, as cannabinoids have demonstrated significant potential in controlling postoperative pain and inflammation [3,41].

Cannabinoids have been increasingly used as part of pain management strategies, particularly for chronic pain conditions such as cancer pain, neuropathic pain (e.g., diabetic neuropathy, multiple sclerosis), orofacial pain [42,43], fibromyalgia, arthritis, and chronic lower back pain [44,45]. Tetrahydrocannabinol (THC) modulates pain perception through CB1 receptor activation, reducing pain transmission in the central nervous system [46]. Cannabidiol (CBD), on the other hand, exerts anti-inflammatory effects by decreasing pro-inflammatory markers such as TNF-α and IL-6 via indirect ECS modulation and interaction with TRPV1 receptors, leading to reduced pain perception [47]. In clinical studies, patients treated with CBD experienced a 30% reduction in postoperative pain within 24 h compared to those treated with NSAIDs, suggesting that cannabinoids provide an effective non-opioid alternative for pain management [48]. However, the administration of cannabidiol (CBD) as therapy for orofacial pain or oral ulcers should aim to achieve a clinical effect with minimal side effects. Both acute and chronic CBD treatments were tested on human oral cells, with acute treatment showing more significant effects than chronic treatment, particularly in fibroblasts. Concentrations of CBD ≥50 μM were found to be highly cytotoxic, inducing apoptosis and reducing cell migration. These findings suggest that further investigation is needed to evaluate the dose- and time-dependent effects of CBD to identify the optimal therapeutic dose. In the study of Umpreecha C. [33], the authors showed that topical 0.1% cannabidiol (CBD) had no allergic reactions or side effects.

Other clinical indications include neurological disorders (e.g., epilepsy, multiple sclerosis, especially for spasticity and muscle spasms, Parkinson’s disease, for motor symptoms and tremors, Alzheimer’s disease), anxiety and mood disorders, and inflammatory and autoimmune diseases (e.g., rheumatoid arthritis, Crohn’s disease, ulcerative colitis, psoriasis) [49,50,51].

Subgroup analysis revealed both consistencies and notable variabilities across the included studies. The analgesic and anti-inflammatory effects of cannabidiol (CBD) were consistently observed across clinical, preclinical, and in vitro models, particularly in managing orofacial pain and periodontitis. However, variability emerged in the context of bone healing, where outcomes depended on the cannabinoid type, delivery route, and study model. For instance, while preclinical studies reported enhanced bone regeneration and reduced resorption following CBD or CB2 receptor agonist administration, clinical evidence remains limited and less conclusive. Moreover, oral and topical routes of cannabinoid delivery demonstrated different degrees of efficacy, with topical applications showing faster localized pain relief and fewer side effects. These findings underscore the importance of stratified analysis to identify which cannabinoid formulations and administration methods are most effective for specific oral conditions, ultimately strengthening the validity and clinical applicability of the overall findings.

Moreover, additional specificity regarding the different types of cannabinoids studied could help contextualize the therapeutic implications more precisely. Among the studies included in this review, cannabidiol (CBD) was the most frequently investigated compound, noted for its non-psychoactive profile and broad anti-inflammatory, analgesic, and regenerative effects. Only one study explored tetrahydrocannabinol (THC) [24]. Synthetic analogs, such as HU-308 (a selective CB2 receptor agonist) and methanandamide (Meth-AEA, a CB1 agonist), were also evaluated in preclinical models, with findings suggesting significant anti-inflammatory and osteoprotective effects. This distinction between phytocannabinoids (CBD, THC) and synthetic analogs (e.g., HU-308, Meth-AEA) is important, as they differ in receptor selectivity, side effect profiles, and potential clinical applications. Greater granularity in future research comparing these agents head-to-head would be valuable in determining their optimal use in dental and oral surgical contexts.

Further studies could focus on CBD’s anti-inflammatory and regenerative properties, as it promotes bone regeneration and accelerates osseointegration by activating CB2 receptors, a topic particularly relevant for dental implantology [52,53]. This activation stimulates osteoblast activity while inhibiting osteoclast-mediated bone resorption. Preclinical studies have shown that CBD accelerates callus formation, enhances bone density, and improves the biomechanical strength of regenerated tissue. Additionally, CBD’s interaction with PLOD1, an enzyme crucial for collagen stabilization, contributes to the mechanical resilience of bone tissue [54]. These findings underscore the significant potential of cannabinoids as adjuvant therapies to improve outcomes in dental implantology and support their broader role in regenerative medicine and surgical applications. In addition, the synergistic effects between cannabinoids, terpenes, and flavonoids, known as the entourage effect, significantly enhance the therapeutic potential of individual compounds [55]. For instance, beta-caryophyllene, a terpene, acts as a CB2 receptor agonist, complementing CBD’s anti-inflammatory properties, while flavonoids like quercetin enhance antioxidant and anti-inflammatory effects, further reducing pain and inflammation. Preclinical models have shown that combining CBD with terpenes, such as myrcene, enhances cellular permeability, improving pain and inflammation control [56]. Additionally, CBD’s anxiolytic effects, mediated through its interaction with serotonin 5-HT1A receptors, help manage preoperative anxiety, which can positively influence surgical outcomes [57]. Clinical studies indicate that CBD treatment can reduce anxiety by up to 40% compared to a placebo, improving patient comfort and facilitating smoother postoperative recovery [58]. These findings highlight CBD’s potential as a holistic therapeutic option in dentistry, While the current findings highlight the promising therapeutic potential of cannabinoids—particularly CBD—in managing orofacial pain, oral inflammation, and promoting bone healing, their immediate integration into dental practice remains premature. Although several preclinical studies have demonstrated consistent analgesic and anti-inflammatory effects, and a few human trials report favorable outcomes with minimal side effects, the overall clinical evidence is still limited by small sample sizes, variability in dosing, cannabinoid formulations, and outcome measures. Furthermore, most randomized controlled trials evaluated short-term effects, and therefore long-term safety, optimal dosing protocols, and drug interactions remain unclear. Therefore, while these results offer a compelling rationale for further research, especially in dental pain and postoperative management, more large-scale, standardized clinical trials are required before cannabinoids can be fully endorsed as routine adjuncts in dental care.

Furthermore, as with every drug, also CBDs caused adverse effects included sedation (calmness or relaxation), diarrhea, and abdominal pain [59]. Nevertheless, they were resolved quickly or with minor interventions. Moreover, chronic treatments with concentrations of CBD ≥ 50 μM were found to be highly cytotoxic, inducing apoptosis and reducing cell migration [60].

This stated the need to evaluate the dose- and time-dependent effects of CBD to identify the optimal therapeutic dose.

This systematic review has several limitations that must be acknowledged. First, the included studies were highly heterogeneous in terms of study design, cannabinoid type (CBD, THC, CB1/CB2 agonists, cannabis smoke), administration route (topical, oral, systemic), and outcome measures. Second, more than half of the studies included were preclinical (animal or in vitro), and nine of the animal studies were judged to have a high risk of bias, weakening the strength of the evidence base. Third, although four randomized controlled trials were included, one was rated as moderate risk of bias and they evaluated different conditions (e.g., acute dental pain, myofascial pain, and oral ulcers), reducing the generalizability of findings. Finally, due to the variability in outcome reporting and the lack of standardized effect size data, meta-analysis could not be performed, limiting quantitative synthesis.

## 5. Conclusions

Within the limitations of this review, cannabinoids—especially cannabidiol (CBD)—demonstrated potential in managing orofacial pain, reducing inflammation, and promoting bone healing. However, these conclusions are drawn from a heterogeneous body of evidence, including multiple preclinical studies with high risk of bias and clinical trials with varying outcomes and endpoints. Notably, studies using different cannabinoid agents (e.g., THC, CB2 agonists, and cannabis smoke) were grouped without sufficient comparative analysis, limiting the strength of overarching conclusions. Therefore, while the therapeutic application of cannabinoids in dentistry is promising, especially as non-opioid alternatives, current evidence does not yet support immediate integration into clinical practice. Rigorous, well-powered, and standardized clinical trials are needed to confirm efficacy, determine safety, and guide dosage and formulation strategies.

## Figures and Tables

**Table 1:** Search strategy.

Database	Search Strategy	Hits
**PubMed**	(((“cannab_*_”[All Fields]) OR (“tetrahydrocannabinol”[All Fields])) AND ((“dentistry”[All Fields]) OR (“dental”[All Fields]) OR (“oral surgery”[All Fields]) OR (“implant_*_”[All Fields]) OR (“toothache”[All Fields]) OR (“periodontitis”[All Fields])) AND ((“pain”[All Fields]) OR (“acute”[All Fields]) OR (“chronic”[All Fields]) OR (“bone”[All Fields]) OR (“bone healing”[All Fields]) OR (“bone regeneration”[All Fields]) OR (“bone resorption”[All Fields])));	449
**Scopus**	TITLE-ABS-KEY (cannab_*_) OR TITLE-ABS-KEY (tetrahydrocannabinol) AND (TI-TLE-ABS-KEY (dentistry) OR TITLE-ABS-KEY (dental) OR TITLE-ABS-KEY (oral AND surgery) OR TITLE-ABS-KEY (implant_*_) OR TITLE-ABS-KEY (toothache) OR TI-TLE-ABS-KEY (periodontitis)) AND (TITLE-ABS-KEY (pain) OR TITLE-ABS-KEY (acute) OR TITLE-ABS-KEY (chronic) OR TITLE-ABS-KEY (bone) OR TITLE-ABS-KEY (bone AND healing) OR TITLE-ABS-KEY (bone AND regeneration) OR TITLE-ABS-KEY (bone AND resorption));	429
**Web of science**	(TS = (cannab_*_) OR TS = (tetrahydrocannabinol_*_)) AND (TS = (dental) OR TS = (oral surgery) OR TS = (implant_*_) OR TS = (toothache) OR TS = (periodontitis)) AND (TS = (pain) OR TS = (acute) OR TS = (chronic) OR TS = (bone) OR TS = (bone healing) OR TS = (bone regeneration) OR TS = (bone resorption));	201

**Table 2:** References excluded and reason for the exclusion.

Reference	Exclusion Criteria
Borsani, E. et al., 2014 [34]	Main topic is related to CBD receptors, not cannabis substances
de Andrade Silva, S. et al., 2024 [35]	No free full text available
Nogueira-Filho, G.R. et al., 2011 [36]	Main topic is related to impact of cannabis smoke on alveolar bone
Ossola, C.A. et al., 2020 [37]	Main topic is related to CBD receptors, not cannabis substances
Qi, X. et al., 2021 [38]	Main topic is related to effects of CBD on human dental pulp cells
Qian, H. et al., 2009 [39]	Narrative review

**Table 3:** Subgroup analysis by condition treated.

Condition	No. of Studies	Cannabinoid Used	Outcomes
Orofacial Pain	5	CBD	Consistent reduction in pain
Oral Inflammation	6	CBD, HU-308	Significant anti-inflammatory effects with reduction in TNF-α, IL-1β
Bone Healing	4	CBD, THC	Enhanced bone density, osteoblast activity

**Table 4:** Subgroup analysis by cannabinoid used.

Cannabinoid	No. of Studies	Outcomes
CBD	10	Analgesic, anti-inflammatory, regenerative effects
THC	1	Delayed tooth movement, reduced bone resorption
HU-308	1	Decreased bone loss and inflammation

**Table 5:** Subgroup analysis by study model.

Study Type	No. of Studies	Outcomes
Clinical	4	Pain relief in human subjects; low side effects
Animal	9	Strong anti-inflammatory, regenerative effects
In Vitro	1	Increased osteogenic markers in human dental pulp cells

**Table 6:** Subgroup analysis by mode of administration.

Administration Type	No. of Studies	Outcomes
Topical	3	Effective for pain and inflammation with minimal side effects
Oral	6	Systemic benefits observed in pain/inflammation
Intraperitoneal	1	Slowed tooth movement in orthodontic model

**Table 7:** Study characteristics.

Author, Year of Publication	Study Design	Intervention	Control	Outcome	Results	Conclusion
**Chen, H. et al. [20]**	Preclinical study in rats	Topical cannabidiol	NR	Anti-inflammatory effects and osteoprotective actions of CBD in periodontitis.	Cannabidiol significantly inhibited bone loss in experimental rat periodontitis models and downregulated the pro-inflammatory mediator TNF-α.	Topical CBD application is effective in treating periodontitis.
**Chrepa, V. et al. [21]**	Research Reports: Clinical study in humans	Oral solution cannabidiol	Placebo	Significant analgesic effect of CBD in dental pain.	Cannabidiol provided a significant reduction in dental pain in humans.	CBD is a safe alternative to opioid or NSAIDs.
**Cohelo, J.C. et al. [22]**	Preclinical study in cats	Oral cannabidiol	Placebo	Anti-inflammatory and analgesic effect of CBD in periodontitis.	Cannabidiol improved levels of comfort and reduced inflammation in cats, without causing adverse effects.	CBD decreases cellular re-uptake of anandamide and FAAH-mediated catabolism, suggesting the benefits of CBD in treating periodontitis.
**Kim, S.W. et al. [23]**	Preclinical study in rats	CBD and taurine combination	Vehicle (untreated periodontitis)	Anti-inflammatory and anti-resorptive effects of CBD and taurine in periodontitis.	Combined CBD and taurine significantly reduced levels of pro-inflammatory mediators, alveolar bone resorption and pocket depth.	The combination of CBD and taurine exhibited synergistic effects in reducing inflammation, bone resorption, and pocket depth, making it a promising therapeutic approach for periodontitis.
**Klein, K.P. et al. [24]**	Animal study in rats	Daily intraperitoneal injections of dronabinol (10 mg/kg) for 21 days during orthodontic tooth movement	Solvent injection	Alveolar bone remodeling and orthodontic tooth movement.	Dronabinol slowed tooth movement, preserved alveolar crest height, and increased osteoblast numbers.	Dronabinol interferes with bone resorption and decelerates orthodontic tooth movement. It should be considered when assessing drug effects on treatment planning.
**Napimoga, M.H. et al. [25]**	Preclinical study in rats	Systemic cannabidiol	Vehicle	Anti-inflammatory and anti-resorptive effects of CBD in periodontitis.	CBD significantly inhibited bone loss and reduced pro-inflammatory cytokines like IL-1β and TNF-α.	CBD demonstrated protective effects in experimental periodontitis, suggesting its potential as a therapeutic agent.
**Nitecka-Buchta, A. et al. [26]**	Clinical study in humans	Cannabidiol formulation for dermal application	Placebo	Myorelaxant and antinociceptive effect.	The surface electromyography masseter muscle activity and the average pain level of masseter muscles were significantly decreased in the test group.	The application of CBD formulation over masseter muscle reduced the activity of masseter muscles and improved the condition of masticatory muscles in patients with myofascial pain.
**Nogueira-Filho Gda, R. et al. [27]**	Preclinical study in rats	Cannabis sativa smoke inhalation	Control group (no smoke exposure)	Impact on bone healing around titanium implants.	MSI reduced cancellous bone-to-implant contact (BIC) and bone area (BA), while no effect was seen in cortical bone.	Cannabis smoke impairs trabecular bone healing around implants, posing a risk to implant success.
**Ossola, C.A. et al. [28]**	Preclinical study in rats	HU-308 (CB2 receptor agonist)	LPS-induced periodontitis without HU-308 treatment	Anti-inflammatory, osteoprotective, and pro-homeostatic effects of HU-308 in periodontitis.	HU-308 treatment significantly reduced alveolar bone loss, pro-inflammatory mediators levels compared to untreated LPS groups. It also restored salivary function.	HU-308 demonstrates therapeutic potential in managing periodontitis and improving salivary function.
**Ossola, C.A. et al. [29]**	Preclinical study in rats	Methanandamide (Meth-AEA), a CB1 receptor agonist	LPS-induced periodontitis model	Anti-inflammatory and osteoprotective effects of Meth-AEA.	Meth-AEA significantly reduced alveolar bone loss and decreased inflammatory markers	Long-term Meth-AEA treatment attenuates LPS-induced periodontitis progression.
**Ostenfeld, T. et al. [30]**	Randomized controlled trial	GW842166 (CB2 receptor agonist)	Placebo and ibuprofen	Analgesic efficacy of GW842166 compared to ibuprofen in dental pain	GW842166 (800 mg) showed trends for pain reduction but failed to demonstrate statistical or clinical significance compared to ibuprofen	GW842166 provided acceptable safety but lacked clinically meaningful efficacy compared to ibuprofen.
**Qi, X. et al. [31]**	Preclinical study in mice	Cannabidiol oral spray	NR	Anti-inflammatory and healing effects of CBD on oral ulcers	CBD oral spray inhibited inflammation, relieved pain, and accelerated lesion closure in acid- or trauma-induced oral ulcers in mice.	CBD accelerates oral ulcer healing, demonstrating therapeutic potential for oral ulcers.
**Qi, X. et al. [32]**	In vitro study on human dental pulp cells	Cannabidiol	NR	Odonto/osteogenic capacity of CBD on HDPCs	CBD promoted HDPC migration, enhanced collagen synthesis, increased mineralized deposits, and upregulated odonto/osteogenic and angiogenic genes.	CBD is a potential agent to develop new therapeutics in vital pulp treatment in dentistry, due to its odonto/osteogenic properties.
**Umpreecha, C. et al. [33]**	Randomized controlled trial	Topical 0.1% CBD	Placebo and 0.1% triamcinolone acetonide (TA)	Efficacy and safety of CBD in managing recurrent aphthous ulcers	CBD significantly reduced ulcer size, erythematous border, and pain levels compared to placebo.	Topical 0.1% CBD reduced ulcer size and accelerated healing without side effects.

**Table 8:** Assessment of quality and risk of bias for randomized controlled trials (RCT) included in the systematic review. Each domain was satisfied (yes), not satisfied (no), unclear, or not assessable (N/A) according to the Joanna Briggs Institute Critical Appraisal tool.

Study	Was True Randomization Used for Assignment of Participants to Treatment Groups?	Was Allocation to Treatment Groups Concealed?	Were Treatment Groups Similar at the Baseline?	Were Participants Blind to Treatment Assignment?	Were Those Delivering Treatment Blind to Treatment Assignment?	Were Outcomes Assessors Blind to Treatment Assignment?	Were Treatment Groups Treated Identically Other Than the Intervention of Interest?	Was Follow-Up Complete and If Not, Were Differences Between Groups in Terms of Their Follow Up Adequately Described and Analyzed?	Were Participants Analyzed in the Groups to Which They Were Randomized?	Were Outcomes Measured in the Same Way for Treatment Groups?	Were Outcomes Measured in a Reliable Way?	Was Appropriate Statistical Analysis Used?	Was the Trial Design Appropriate, and Any Deviations from the Standard RCT Design (Individual Randomization, Parallel Groups) Accounted for in the Conduct and Analysis of the Trial?	Overall Risk of Bias
**Chrepa,** V. et al. [21]	YES	YES	YES	YES	YES	YES	YES	YES	YES	YES	YES	YES	YES	Low
**Nitecka-Buchta,** A. et al. [26]	YES	YES	YES	YES	YES	YES	YES	YES	YES	YES	YES	YES	YES	Low
**Ostenfeld,** T. et al. [30]	YES	YES	YES	YES	YES	UNCLEAR	YES	YES	YES	YES	YES	YES	YES	Moderate
**Umpreecha,** C. et al. [33]	YES	YES	YES	YES	YES	YES	YES	YES	YES	YES	YES	YES	YES	Low

**Table 9:** Summary of risk of bias assessment according to SYRCLE’s risk of bias for studies in animals.

Chen, H. et al. [20]	Type of Bias		Domain	Description of Domain	Review Author Judgment
	Selection bias		Sequence generation	Not reported	High risk
	Selection bias		Baseline characteristics	The groups were similar at baseline	Low risk
	Selection bias		Allocation concealment	Not reported	High risk
	Performance bias		Random housing	Not reported	High risk
	Performance bias		Blinding	Not reported	High risk
	Detection bias		Random outcome assessment	Not reported	High risk
	Detection bias		Blinding	Not reported	High risk
	Attrition bias		Incomplete outcome data	Not reported	High risk
	Reporting bias		Selective outcome reporting	Not reported	High risk
	Other		Other sources of bias	The study appears to be free of other sources of bias	Low risk
**Cohelo, J.C. et al. [22]**	**Type of Bias**		**Domain**	**Description of Domain**	**Review Author Judgment**
	Selection bias		Sequence generation	Not reported	High risk
	Selection bias		Baseline characteristics	The groups were similar at baseline	Low risk
	Selection bias		Allocation concealment	Not reported	High risk
	Performance bias		Random housing	Not reported	High risk
	Performance bias		Blinding	The owners were not aware if their animal was receiving CBD formulation or a placebo	Low risk
	Detection bias		Random outcome assessment	Not reported	High risk
	Detection bias		Blinding	Not reported	High risk
	Attrition bias		Incomplete outcome data	Not reported	High risk
	Reporting bias		Selective outcome reporting	Not reported	High risk
	Other		Other sources of bias	The study appears to be free of other sources of bias	Low risk
**Kim, S.W. et al. [23]**	**Type of Bias**		**Domain**	**Description of Domain**	**Review Author Judgment**
	Selection bias		Sequence generation	Not reported	High risk
	Selection bias		Baseline characteristics	The groups were similar at baseline	Low risk
	Selection bias		Allocation concealment	Not reported	High risk
	Performance bias		Random housing	Not reported	High risk
	Performance bias		Blinding	Not reported	High risk
	Detection bias		Random outcome assessment	Not reported	High risk
	Detection bias		Blinding	Not reported	High risk
	Attrition bias		Incomplete outcome data	Not reported	High risk
	Reporting bias		Selective outcome reporting	Not reported	High risk
	Other		Other sources of bias	The study appears to be free of other sources of bias	Low risk
**Klein, K.P. et al. [24]**	**Type of Bias**		**Domain**	**Description of Domain**	**Review Author Judgment**
	Selection bias		Sequence generation	Not reported	High risk
	Selection bias		Baseline characteristics	The groups were similar at baseline	Low risk
	Selection bias		Allocation concealment	Not reported	High risk
	Performance bias		Random housing	Not reported	High risk
	Performance bias		Blinding	Not reported	Low risk
	Detection bias		Random outcome assessment	Not reported	High risk
	Attrition bias		Incomplete outcome data	Not reported	High risk
	Reporting bias		Selective outcome reporting	Not reported	High risk
	Other		Other sources of bias	The study appears to be free of other sources of bias	Low risk
**Napimoga, M.H. et al. [25]**		**Type of Bias**	**Domain**	**Description of Domain**	**Review Author Judgment**
		Selection bias	Sequence generation	Not reported	High risk
		Selection bias	Baseline characteristics	The groups were similar at baseline	Low risk
		Selection bias	Allocation concealment	Not reported	High risk
		Performance bias	Random housing	Not reported	High risk
		Performance bias	Blinding	Not reported	High risk
		Detection bias	Random outcome assessment	Not reported	High risk
		Detection bias	Blinding	Immunohistochemical analysis was performed individually by two examiners who were blind to the treatment conditions	Low risk
		Attrition bias	Incomplete outcome data	Not reported	High risk
		Reporting bias	Selective outcome reporting	Not reported	High risk
		Other	Other sources of bias	The study appears to be free of other sources of bias	Low risk
**Nogueira-Filho Gda, R. et al. [27]**		**Type of Bias**	**Domain**	**Description of Domain**	**Review Author Judgment**
		Selection bias	Sequence generation	Not reported	High risk
		Selection bias	Baseline characteristics	The groups were similar at baseline	Low risk
		Selection bias	Allocation concealment	Not reported	High risk
		Performance bias	Random housing	Not reported	High risk
		Performance bias	Blinding	The owners were not aware if their animal was receiving CBD formulation or a placebo	Low risk
		Detection bias	Random outcome assessment	Not reported	High risk
		Detection bias	Blinding	The percentage of bone-to-implant contact and bone area within the threads of the implants were obtained by a blinded examiner	Low risk
		Attrition bias	Incomplete outcome data	Not reported	High risk
		Reporting bias	Selective outcome reporting	Not reported	High risk
		Other	Other sources of bias	The study appears to be free of other sources of bias	Low risk
**Ossola** **, C** **.A** **. et al. [28]**		**Type of Bias**	**Domain**	**Description of Domain**	**Review Author Judgment**
		Selection bias	Sequence generation	Not reported	High risk
		Selection bias	Baseline characteristics	The groups were similar at baseline	Low risk
		Selection bias	Allocation concealment	Not reported	High risk
		Performance bias	Random housing	Not reported	High risk
		Performance bias	Blinding	Not reported	High risk
		Detection bias	Random outcome assessment	Not reported	High risk
		Detection bias	Blinding		
		Attrition bias	Incomplete outcome data	Not reported	High risk
		Reporting bias	Selective outcome reporting	Not reported	High risk
		Other	Other sources of bias	The study appears to be free of other sources of bias	Low risk
**Ossola, C.A. et al. [29]**	**Type of Bias**		**Domain**	**Description of Domain**	**Review Author Judgment**
	Selection bias		Sequence generation	Not reported	High risk
	Selection bias		Baseline characteristics	The groups were similar at baseline	Low risk
	Selection bias		Allocation concealment	Not reported	High risk
	Performance bias		Random housing	Not reported	High risk
	Performance bias		Blinding	Not reported	Low risk
	Detection bias		Random outcome assessment	Not reported	High risk
	Detection bias		Blinding	Not reported	High risk
	Attrition bias		Incomplete outcome data	Not reported	High risk
	Reporting bias		Selective outcome reporting	Not reported	High risk
	Other		Other sources of bias	The study appears to be free of other sources of bias	Low risk
**Qi, X. et al. [31]**	**Type of Bias**		**Domain**	**Description of Domain**	**Review Author Judgment**
	Selection bias		Sequence generation	Not reported	High risk
	Selection bias		Baseline characteristics	The groups were similar at baseline	Low risk
	Selection bias		Allocation concealment	Not reported	High risk
	Performance bias		Random housing	Not reported	High risk
	Performance bias		Blinding	The experiments and assessment of outcomes were performed in a blinded manner	Low risk
	Detection bias		Random outcome assessment	Not reported	High risk
	Detection bias		Blinding	Clinical inflammatory evaluation was conducted in a single-blind manner Behavior analysis was performed by an investigator who was blinded to the group assignment	Low risk
	Attrition bias		Incomplete outcome data	Not reported	High risk
	Reporting bias		Selective outcome reporting	Not reported	High risk
	Other		Other sources of bias	The study appears to be free of other sources of bias	Low risk

**Table 10:** Assessment of risk of bias for in vitro dental studies according to QUIN tool.

Study	Criteria	Adequately Specified (Score = 2)	Inadequately Specified (Score = 1)	Not Specified (Score = 0)	Not Applicable
**Qi** **, X. et al. [32]**	Clearly stated aims/objectives	X			
	Detailed explanation of sample size calculation			X	
	Detailed explanation of sampling technique	X			
	Details of comparison group				X
	Detailed explanation of methodology	X			
	Operator details			X	
	Randomization				X
	Method for measurement of outcome	X			
	Outcome assessor details	X			
	Blinding				X
	Statistical analysis	X			
	Presentation of results	X			

## Data Availability

The data are included in the manuscript.
